# COVID-19 Vaccine-Related Arthritis: A Descriptive Study of Case Reports on a Rare Complication

**DOI:** 10.7759/cureus.26702

**Published:** 2022-07-09

**Authors:** Rand Dawoud, Daniel Haddad, Viraj Shah, Vraj Patel, Gohar Abbas, Sai Guduru, Amulya Dakka, Vishrut Kaushik, Pramil Cheriyath

**Affiliations:** 1 Medicine, The Hashemite University, Amman, JOR; 2 Internal Medicine, Rowan University School of Osteopathic Medicine, Glassboro, USA; 3 Internal Medicine, Hackensack Meridian Ocean University Medical Center, Brick, USA; 4 Internal Medicine, Rajarshee Chhatrapati Shahu Maharaj Government Medical College, Kolhapur, IND; 5 Internal Medicine, AUA School of Medicine, Brick, USA; 6 Internal Medicine, North American Dental Group, Brick, USA; 7 Internal medicine, Mosaic Life Care, Saint Joseph, USA

**Keywords:** joint-related adverse effect, mrna-based vaccine, arthritis, covid-19 vaccine, covid-19

## Abstract

Large-scale coronavirus disease 2019 (COVID-19) vaccination programs have been rolled out worldwide. Vaccines that are widely used globally include mRNA vaccines, adenoviral vector vaccines, and inactivated whole-virus vaccines. COVID-19 vaccines can lead to varying side effects. Among the most common of these adverse effects are pain at the injection site, fatigue, and headaches. Some side effects, however, are not very well documented, and these include joint-related adverse effects. In this review, we assess the epidemiology and clinical features of post-COVID-19 vaccination joint-related adverse effects based on the analysis of 16 patient case reports.

Based on our analysis, we found that females formed the majority of the cases, accounting for 62.5% of patients, while 37.5% of the cases were males. The mean age of presentation among the patients was 54.8 years, with a standard deviation (SD) of 17.49 years. In 37.5% of the cases, patients received the Sinovac vaccine. The proportion of patients who received other vaccines was as follows: the Pfizer vaccine: 31.25%; Sputnik V: 12.5%; Moderna, AstraZeneca, and Covaxin: 6.25% each.

The characteristics of joint-related adverse effects following COVID-19 vaccination were analyzed in this study. We identified several key findings related to factors such as age, gender, type of vaccine, clinical features, and diagnosis modality. Our analysis showed that more cases were reported among individuals who received the Sinovac vaccine, as compared to the others. Further research is required to examine the underlying cause of this association.

## Introduction and background

Various coronavirus disease 2019 (COVID-19) vaccines have been developed at a remarkably accelerated pace, with more than nine vaccines already having official approval for use around the world [1,2]. These primarily include mRNA vaccines (Pfizer, Moderna), adenoviral vector vaccines (AstraZeneca, Johnson Pharma), and inactivated whole-virus vaccines (Sinopharm, Sinovac) [3]. Three billion doses of the COVID-19 vaccine have been administered globally so far, and at least 5% of the global population had received their first dose of vaccination by mid-2021 [3,4]. COVID-19 vaccines have played a critical role in effectively reducing the rates of COVID-19 infection, transmission, hospitalizations, and deaths worldwide [5]. However, like all vaccines, COVID-19 vaccines can have varying side effects, ranging from mild pain at the site of injection [6] to myocarditis [7] and even thrombosis [8]. Nevertheless, not all side effects of these vaccinations have been detected in clinical trials, and hence their recognition is of utmost clinical importance. With the commencement of large-scale vaccination programs across the world, several case reports have linked these vaccinations to joint-related adverse effects, including new-onset arthritis, joint disease flare-ups, and frank joint injuries. This necessitates a comprehensive assessment of potential joint-related adverse effects associated with COVID-19 vaccination. In light of this, we performed a descriptive study on post-COVID-19 joint-related adverse effects to examine their epidemiology, clinical characteristics, and manifestations.

## Review

Methods

Eligibility Criteria 

We included case reports and case series that dealt with joint-related adverse effects following COVID-19 vaccination with no demographic restrictions; the studies were included regardless of the type of vaccination, duration between the doses of vaccination, and the onset of joint-related symptoms or their severity. Studies that discussed all other COVID-19 vaccine-related adverse events except joint-related ones were excluded. After excluding duplications and applying the inclusion-exclusion criteria, we included 14 published articles in our study, consisting of 16 case reports (Table 1) of joint-related adverse effects after receiving the COVID-19 vaccine. The vaccines involved were Sinovac, Pfizer, Sputnik-V, Moderna, AstraZeneca, and Covaxin.

**Table 1:** Description of case studies included in this review article

No.	Author	Summary
1.	Wharton et al. [9]	A 31-year-old man developed left shoulder pain and limited range of motion 2.5 weeks after the administration of the second dose of the Moderna vaccine
2.	Massel et al. [10]	A 68-year-old woman developed left shoulder glenohumeral joint septic arthritis within one week of receiving the Pfizer vaccine
3.	Terracina et al. [11]	A 55-year-old man, in sustained clinical remission for over two years, developed an acute flare of rheumatoid arthritis affecting his right knee 12 hours after receiving the second dose of the Pfizer vaccine
4.	Baimukhamedov et al. [12]	A 38-year-old woman developed de novo rheumatoid arthritis; with symmetric polyarthritis of the hand, ankle, knee, and shoulder joints three weeks after receiving the first dose of the Sputnik V vaccine
5.	Cantarelli Rodrigues et al. [13]	A 61-year-old woman developed right subacromial-subdeltoid bursitis 30 minutes after receiving her first dose of the AstraZeneca vaccine. An erroneous injection into the shoulder bursa may have precipitated this
6.	Chuaychoosakoon et al. [14]	A 52-year-old man developed right subacromial-subdeltoid bursitis three days after receiving a Sinovac injection
7.	An et al. [15]	A 23-year-old woman developed acute reactive arthritis of her left knee one week after Sinovac administration, with remission and recurrence two days after the second dose was given on day 14
8, 9.	Unal Enginar et al. [16]	Case 1: a 74-year-old woman developed arthritis in her right wrist, metacarpophalangeal, and proximal interphalangeal joints two days after the first dose of Sinovac. Case 2: a 76-year-old man developed arthritis of the left hand one week following the second dose of Sinovac
10.	Baimukhamedov et al. [17]	A 58-year-old man developed left elbow joint arthritis seven days after the administration of the second dose of Sputnik V
11.	Park et al. [18]	A 36-year-old woman developed adult-onset Still’s disease 10 days after the first dose of Pfizer, with bilateral polyarthritis of the hands and ankles
12, 13.	Sharabi et al. [19]	Case 1: a 43-year-old man developed adult-onset Still’s disease 10 days after receiving the second dose of Pfizer, with bilateral knee joint involvement. Case 2: a 56-year-old woman developed adult-onset Still’s disease 10 days after receiving the second dose of Pfizer, with bilateral hand, knee, and ankle joint involvement
14.	Singh et al. [20]	A woman in her late 50s developed de novo rheumatoid arthritis with refractory reactive eosinophilia less than two weeks after receiving the first dose of Covaxin, with a bilateral elbow, wrist, metacarpophalangeal, and proximal interphalangeal joint involvement
15, 16.	Türk et al. [21]	Case 1: a 72-year-old woman developed reactive polyarthritis of the left elbow, bilateral knees, and right ankle 21 days after receiving the first dose of Sinovac. Case 2: a 79-year-old woman developed reactive polyarthritis of the bilateral hand joints and left ankle joint five days after receiving the second dose of Sinovac

Search Strategies

A literature search using relevant keywords was performed through Google and scientific databases such as Google Scholar and PubMed to identify case reports on post-COVID-19 vaccination-related joint adverse effects. We used keywords like COVID-19, COVID-19 vaccination, mRNA-based vaccine, joint-related adverse effects, and arthritis. The literature search sought out all relevant articles published between the period of November 2020 and March 2022. After considering all the inclusion and exclusion criteria, 16 case reports were finalized for this study.

Data Collection Process and Data Items

Data were extracted independently by two authors by using standardized data extraction forms. We documented characteristics like age, gender, and other variables such as the type of vaccination, signs/symptoms, date of diagnosis, methods of diagnosis outcome, laboratory values, and results on an Excel sheet (Microsoft Corporation, Redmond, WA). These variables were then analyzed.

Statistical Analysis

Patient demographic characteristics, disease manifestations, and causes were summarized descriptively and analyzed using R version 1.1.456 (RStudio: Integrated Development for R. RStudio PBC, Boston, MA).

Results

In this study of 16 case reports on joint-related adverse effects linked to COVID-19 vaccination, the average age of presentation was 54.8 ± 17.49 years. Of all reported cases, 10 (62.5%) were females, and six (37.5%) were males. Ethnicity was split evenly among cases, with six (37.5%) patients being of Asian descent and six (37.5%) of Caucasian descent.

Of the 16 cases, Sinovac vaccine administration was reported in most of the arthritis cases, accounting for six (37.5%) cases. Pfizer was reported in five cases (31.25%), whereas two cases (12.5%) received Sputnik-V. One case (6.25%) each of arthritis was reported in patients receiving Moderna, AstraZeneca, and Covaxin vaccines. Symptom onset was observed in half of all cases after the second dose, with an average duration of onset after 7.38 days and all reported cases occurring within two and a half weeks. Symptoms were seen after an average of 11.34 days in cases related to the first dose, which made up 37.5% of total cases. Similarly, all cases occurred within three weeks after receiving the vaccine. 

Joint involvement varied widely among cases. Half of all cases involved a single joint [monoarticular, eight cases (50%)]. This included all joints listed except the ankle. Likewise, polyarticular arthritis was seen in eight cases (50%). On further review of specific joint involvement, it was observed that the joints of the hand, such as the metacarpophalangeal (MCP), proximal interphalangeal (PIP), and distal interphalangeal (DIP) joints, comprised the majority of cases [7/16 (43.75%)]. This was followed by cases involving the knee with six cases (37.5%), shoulder and ankle with five cases (31.25%), and elbow joints with three cases (18.75%).

In most cases, two non-specific markers of inflammation - erythrocyte sedimentation rate (ESR) and C-reactive protein (CRP) - were monitored. The average ESR was elevated at 57.3 mm/hr with an SD of 29.09 mm/hr; likewise, the average CRP was elevated at 12.33 mg/dL with an SD of 10.46 mg/dL. Arthrocentesis of affected joint(s) was performed in five cases (31.25%).

Clinical remission was reported in 11 of the 16 cases (68.75%). Unfavorable outcomes were not reported in any of the cases in this review, with five cases not reporting data related to patient follow-up (Table 2).

**Table 2:** Summary of parameters, vaccine characteristics, joint involvement, laboratory values, and recovery SD: standard deviation; ESR: erythrocyte sedimentation rate; CRP: C-reactive protein

Variables	Values
Parameter	
Age, mean ± SD	54.8 ± 17.49 years
Gender, % (n)	
Female	62.5% (10 cases)
Male	37.5% (6 cases)
Ethnicity, % (n)	
Asian	37.5% (6 cases)
Caucasian	37.5% (6 cases)
Unknown	25% (4 cases)
Vaccine characteristics	
Types of vaccination associated, % (n)	
Sinovac	37.5% (6 cases)
Pfizer	31.25% (5 cases)
Sputnik V	12.5% (2 cases)
AstraZeneca	6.25% (1 case)
Covaxin	6.25% (1 case)
Moderna	6.25% (1 case)
Symptom onset after which dose of vaccination? % (n)	
First dose	37.5% (6 cases)
Second dose	50% (8 cases)
Unknown	12.5% (2 cases)
Average duration from vaccination to symptom onset, mean ± SD	
First dose	11.34 ± 9.07 days
Second dose	7.375 ± 5.32 days
Joint involvement, % (n)	
Hand	43.75% (7 cases)
Knee	37.5% (6 cases)
Shoulder	31.25% (5 cases)
Ankle	31.25% (5 cases)
Elbow	18.75% (3 cases)
Monoarticular	50% (8 cases)
Polyarticular	50% (8 cases)
Laboratory values, mean ± SD	
ESR	57.3 ± 29.09 mm/hr
CRP	12.33 ± 10.46 mg/dL
Arthrocentesis performed	31.25% (5 cases)
Recovery, % (n)	
Clinical remission	68.75% (11 cases)
Unknown	31.25% (5 cases)

Discussion

Since the emergence of COVID-19 in November 2019, tens of millions of cases have been reported globally [22]. In an effort to contain the virus and its spread, which has had myriad consequences, mass and targeted vaccination programs have been rolled out worldwide since December 2020 [23]. Since the introduction of COVID-19 vaccines, evidence has shown a notable reduction in virus contagiousness and spread among vaccinated people [24].

COVID-19 vaccines currently in widespread use include mRNA, adenoviral vector, and inactivated whole-virus vaccines, which all depend on the native viral spike (S) protein for generating potent neutralizing antibodies; however, they differ markedly in the presentation of the principal antigen to the immune system [25]. Genetic vaccines, i.e., mRNA and adenoviral vaccines, essentially function by providing mRNA for the biosynthesis of S protein after intramuscular administration. In contrast, inactivated whole-virus vaccines consist of S protein in distinct forms along with adjuvants, primarily aluminum hydroxide [26].

COVID-19 vaccines can have varying side effects, ranging from mild to severe [26]. The most commonly documented adverse effects are local injection site events, such as redness, pain, and swelling [27]. Systemic side effects can also occur, including fever, fatigue, chills, myalgia, arthralgia, and headaches [26,27]. Although rare, certain serious adverse effects have been reported as well, such as myocarditis [28], hypersensitivity reactions, thrombocytopenia, and thrombosis [29].

Less commonly reported, however, are joint-related side effects, including new-onset arthritis, arthralgias, joint disease flare-up, and bursitis. While the pathophysiology of these events is poorly understood, it is hypothesized that vaccines that contain inactivated viral pathogens or attenuated pathogens may function as agents that trigger autoimmune disease [16]. This may have been the case with the inactivated vaccines studied (Covaxin and Sinovac) or the viral vectors mentioned (AstraZeneca and Sputnik V). An autoimmune response with the mRNA vaccines studied (Moderna and Pfizer) may indicate similar mechanisms triggered by viral antigens produced by host cells. In addition, adjuvants in vaccines could elicit a certain autoimmune adverse effect termed “autoimmune/inflammatory syndrome induced by adjuvants (ASIA)” [30]. Two main theories have been proposed to elucidate this development of autoimmunity: one is antigen-specific, an example of which is molecular mimicry, and the latter is known as “bystander activation” [30]. Molecular mimicry occurs when sequence similarities between self and foreign peptides lead to the cross-activation of auto-reactive T or B cells by pathogen-derived antigens in a susceptible individual [31]. Bystander activation refers to auto-reactive T or B cells that activate independently of the presence of an antigen [32]. Of the cases mentioned in this study, only those that involved inactivated vaccines, Sinovac and Covaxin, explicitly report using an adjuvant, both of which are aluminum-based [33]. However, the connection between aluminum adjuvants and ASIA is unclear; one large pharmacoepidemiological study of more than 18,000 patients found a lower incidence of ASIA using aluminum adjuvants compared with controls [34]. In addition, shoulder injury related to vaccine administration (SIRVA) can result from incorrect anatomic vaccine administration to the subacromial space rather than intramuscularly in the deltoid muscle [9].

Patients with COVID-19 vaccination-related new-onset arthritis, arthralgias, joint disease flare-up, and bursitis usually present with swelling, pain, stiffness, and, occasionally, decreased range of motion of the affected joint [9,15,16,35]. The majority of cases in this study were reported within a week of receiving the second dose of the COVID-19 vaccine.

To establish a diagnosis, it could be of value to obtain serum CRP and ESR levels, as they were found to be elevated in most of the reported cases. Arthrocentesis of the joint was performed in select cases in order to characterize the affected joint and distinguish between different etiologies, such as osteoarthritis, gout, and septic arthritis. Furthermore, imaging was also considered in some patients to delineate the pathology further and rule out anatomical defects or trauma. As for treatment, oral and intra-articular corticosteroids were given in the majority of arthritis cases [11,12,16], which resulted in clinical remission in all follow-up appointments reported.

## Conclusions

A literature search performed between November 2020 and March 2022 elicited 16 cases of joint-related adverse effects reported globally among individuals after receiving various COVID-19 vaccine formulations. Although not yet frequently reported, joint-related adverse effects following COVID-19 vaccination can definitely occur. Cases were incidentally split equally among monoarticular and polyarticular arthritis, with the hand and knee being the most involved joints. All cases occurred within three weeks of vaccine administration. The treatment for a majority of cases was similar to that for autoimmune flare-ups, with oral or intra-articular corticosteroids leading to the remission of symptoms within one month in all reported follow-ups. Healthcare workers should be vigilant about all possible differentials in patients with joint pain, swelling, and stiffness that occur within a week following the COVID-19 vaccination. Early diagnosis and prompt treatment can help avert severe consequences and facilitate timely recovery. Further studies are required to gain a better understanding of the associations and mechanisms of these adverse effects due to COVID-19 vaccination.
